# Human DUS1L catalyzes dihydrouridine modification at tRNA positions 16/17, and *DUS1L* overexpression perturbs translation

**DOI:** 10.1038/s42003-024-06942-8

**Published:** 2024-10-02

**Authors:** Jin Matsuura, Shinichiro Akichika, Fan-Yan Wei, Tsutomu Suzuki, Takahiro Yamamoto, Yuka Watanabe, Leoš Shivaya Valášek, Akitake Mukasa, Kazuhito Tomizawa, Takeshi Chujo

**Affiliations:** 1 Department of Molecular Physiology, Faculty of Life Sciences, Kumamoto University, Kumamoto, Japan; 2 Department of Neurosurgery, Faculty of Life Sciences, Kumamoto University, Kumamoto, Japan; 3 Department of Chemistry and Biotechnology, Graduate School of Engineering, University of Tokyo, Tokyo, Japan; 4 Department of Modomics Biology and Medicine, Institute of Development, Aging and Cancer, Tohoku University, Sendai, Japan; 5 Department of Cell Pathology, Faculty of Life Sciences, Kumamoto University, Kumamoto, Japan; 6 Laboratory of Regulation of Gene Expression, Institute of Microbiology of the Czech Academy of Sciences, Prague, Czech Republic; 7 Center for Metabolic Regulation of Healthy Aging, Faculty of Life Science, Kumamoto University, Kumamoto, Japan

**Keywords:** RNA, Chemical modification

## Abstract

Human cytoplasmic tRNAs contain dihydrouridine modifications at positions 16 and 17 (D16/D17). The enzyme responsible for D16/D17 formation and its cellular roles remain elusive. Here, we identify DUS1L as the human tRNA D16/D17 writer. *DUS1L* knockout in the glioblastoma cell lines LNZ308 and U87 causes loss of D16/D17. D formation is reconstituted in vitro using recombinant DUS1L in the presence of NADPH or NADH. *DUS1L* knockout/overexpression in LNZ308 cells shows that DUS1L supports cell growth. Moreover, higher *DUS1L* expression in glioma patients is associated with poorer prognosis. Upon vector-mediated *DUS1L* overexpression in LNZ308 cells, 5′ and 3′ processing of precursor tRNA^Tyr(GUA)^ is inhibited, resulting in a reduced mature tRNA^Tyr(GUA)^ level, reduced translation of the tyrosine codons UAC and UAU, and reduced translational readthrough of the near-cognate stop codons UAA and UAG. Moreover, *DUS1L* overexpression increases the amounts of several D16/D17-containing tRNAs and total cellular translation. Our study identifies a human dihydrouridine writer, providing the foundation to study its roles in health and disease.

## Abstract (web summary)

DUS1L was identified as the human tRNA dihydrouridine writer for positions 16 and 17. DUS1L overexpression changes the amount of several D16/D17-containing tRNAs and increases total cellular translation and cell growth in glioblastoma cells.

## Introduction

tRNA is the adapter molecule that physically links genetic information transcribed in mRNA to proteins^1,2^. tRNAs are post-transcriptionally decorated with a variety of chemical modifications that are incorporated by specific modifying enzymes^3^. These modifications maintain tRNA structural integrity, biochemical stability, and/or appropriate codon–anticodon interactions^3^. Human tRNAs collectively contain more than 40 modifications at specific positions^4^. The importance of these modifications is emphasized by the existence of more than 50 human tRNA modification enzymes with pathogenic mutations^3,5,6^. Deficiency of these enzymes frequently manifests as brain dysfunction, diabetes, short stature, and mitochondrial diseases. On the other hand, high expression of tRNA modification enzymes is frequently associated with cancer progression and is presumed to support cancer growth because knockout (KO) of these enzymes slows the growth of cancer cells in culture dishes and xenografts^5–7^.

**Fig. 1:**
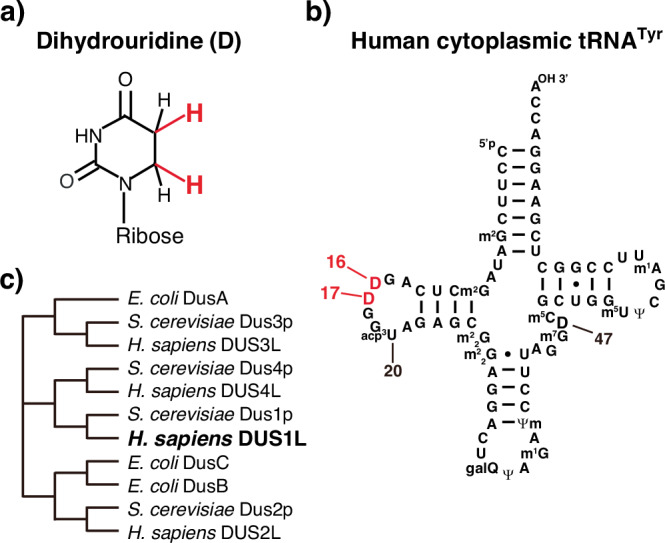
D modifications at positions 16 and 17 of human cytoplasmic tRNAs. **a** Chemical structure of D. **b** Secondary structure of human cytoplasmic tRNA^Tyr(GUA)^ with modified nucleosides: 2-methylguanosine (m^2^G), 3-(3-amino-3-carboxypropyl)uridine (acp^3^U), *N*^2^,*N*^*2*^-dimethylguanosine (m^2^_2_G), galactosyl-queuosine (galQ), pseudouridine (Ψ), *N*^*1*^-methylguanosine (m^1^G), 2’-*O*-methylpseudouridine (Ψm), 7-methylguanosine (m^7^G), 5-methylcytidine (m^5^C), 5-methyluridine (m^5^U), and *N*^*1*^-methyladenosine (m^1^A). Nucleoside positions are numbered following conventional guidelines^61^. Modified nucleosides are depicted according to a previous study^60^. **c** Neighbor-joining tree of DUS homologs from *E. coli*, *S. cerevisiae*, and *H. sapiens* generated by alignment of protein sequences and depiction of a cladogram using Clustal omega^62^ and its associated applications. The DUS homologs and their RefSeq numbers are as follows: *E. coli* DusA (NP_418473.3), DusB (NP_417726.1), and DusC (NP_416645.1); *S. cerevisiae* Dus1p (NP_013631.1), Dus2p (NP_014412.1), Dus3p (NP_013505.4), and Dus4p (NP_013509.1); and *H. sapiens* DUS1L (NP_071439.3), DUS2L (NP_001258691.1), DUS3L (NP_064560.2), and DUS4L (NP_853559.1).

Dihydrouridine (D) is a tRNA modification (Fig. 1a, b) conserved in all three domains of life^8^. It is found at tRNA positions 16, 17, 20, 20a, and 20b in the eponym D-loop and at position 47 in the variable loop^8^. D is formed by addition of two hydrogens at positions 5 and 6 of the uracil base, breaking the C=C double bond between positions 5 and 6 (Fig. 1a). The hydrogen additions remove the aromaticity and planar structure of the uracil base, destabilizing the C3′-endo ribose conformation and allowing less stacking of the base^9–11^. Thus, the D modification provides more conformational flexibility to the D-loop. Consistently, more D is found in psychrophilic bacteria and less D is observed in thermophilic archaea, likely reflecting the need to increase and decrease tRNA flexibility in order to adapt to environmental temperatures, respectively^12,13^.

The dihydrouridine synthase (DUS) family was first discovered in *Escherichia coli* and *Saccharomyces cerevisiae*^14–16^. In eubacteria, three DUS proteins have been identified, each of which is responsible for modifications at specific positions: DusA for D20 and D20a^17–19^, DusB for D17^17^, and DusC for D16^20^. In *S. cerevisiae*, four DUS proteins have been identified: Dus1p for D16 and D17, Dus2p for D20, Dus3p for D47, and Dus4p for D20a and D20b^15^. Biochemical and structural analyses revealed that DUS is a flavin mononucleotide-dependent enzyme that utilizes NADPH for hydrogen addition to the uracil base^16,18,19,21^. Yeast Dus2p requires other tRNA modifications for its activity, suggesting that Dus2p-mediated D formation is a later step in tRNA maturation^22^. Bacterial DusA, DusB, and DusC and the human DUS2L introduced below, commonly recognize the L-shaped structure of tRNA around the “elbow” region, but differ in recognition of tRNAs based on their different orientations, rotation of the target uracil, or use of specific tRNA recognition domains^17,20,23–26^.

Corresponding to yeast Dus1–4p, human has four DUS homologs, which were tentatively named DUS1-like (DUS1L) to DUS4-like (DUS4L) in databases. Similar to yeast Dus2p, human DUS2L is responsible for the tRNA D20 modification^24^. *DUS2L* is highly expressed in non-small cell lung carcinoma (NSCLC) tissues, and high *DUS2L* expression in NSCLC is associated with poor prognosis. *DUS2L* knockdown in a NSCLC cell line reduces proliferation rates. These data suggest that human DUS2L supports pulmonary carcinogenesis^27^. Human DUS1L, DUS3L, and DUS4L are necessary for D modification at tRNA positions 16, 47, and 20a, respectively^28,29^. However, these studies did not investigate the sufficiency of D modification enzymes. In addition, the D modification enzymes responsible for modifications at positions 17 and 20b remain unclear.

In this study, by performing *DUS1L* KO and in vitro reconstitution of D, we show that human DUS1L is responsible for D modifications at tRNA positions 16 and 17. *DUS1L* supported growth of a glioblastoma (WHO grade IV) cell line, and higher *DUS1L* expression was associated with poorer prognosis in glioma patients. Moreover, vector-mediated *DUS1L* overexpression inhibited 5′ and 3′ processing of the tRNA^Tyr(GUA)^ precursor and reduced the levels of mature tRNA^Tyr^ and corresponding UAN codon translation. *DUS1L* overexpression also increased the steady-state levels of several DUS1L-target tRNAs and the overall cellular translation level.

## Results

### *DUS1L* is required for D16 and D17 formation in human cytoplasmic tRNAs

**Fig. 2:**
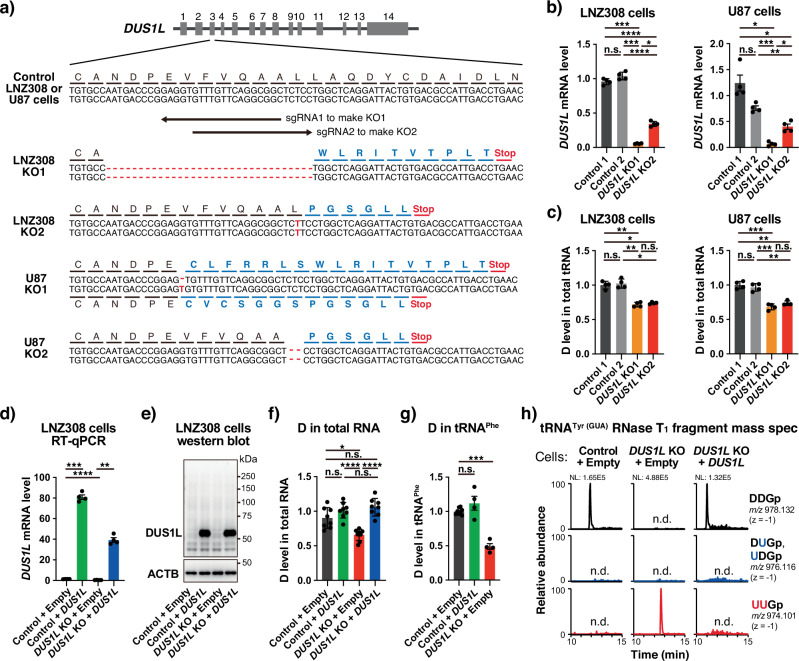
Loss of D16 and D17 modifications in *DUS1L* KO human cells. **a** *DUS1L* alleles in *DUS1L* KO LNZ308 and U87 glioblastoma cell lines. Red lines indicate deletions and red letters indicate insertions. Aberrant amino acids generated by frameshifting are indicated in blue. *DUS1L* sgRNA 1 was used to generate LNZ308 and U87 KO1 cells. *DUS1L* sgRNA 2 was used to generate LNZ308 and U87 KO2 cells. Control cells were generated using sgRNAs that did not target the human genome. **b** RT-qPCR analysis of *DUS1L* mRNA levels normalized by *GAPDH* mRNA levels. Means ± s.e.m. from *n* = 4 samples each. **c** LC-MS analysis of total tRNA nucleosides generated by nuclease P_1_ digestion of total tRNA that was gel-excised after electrophoresis of total RNA. The values are the relative levels to the mean D levels of control 1 and control 2 cells, normalized by the m^1^G peak area of the same samples. Means ± s.e.m. from *n* = 4 samples each. **d** RT-qPCR analysis of *DUS1L* mRNA levels normalized by *GAPDH* mRNA levels in control 1 or *DUS1L* KO2 LNZ308 cells transduced with a lentivirus for stable expression of PAM-mutated synonymous *DUS1L* or with an empty control lentivirus. Means ± s.e.m. from *n* = 4 samples each. **e** Western blot analysis of lysates from the indicated cells using antibodies against DUS1L and ACTB. Endogenous DUS1L was not detected in control cells by western blotting, presumably due to its low expression, similar to many other mammalian tRNA modification enzymes^56^. **f** LC-MS analysis of total RNA nucleosides generated by nuclease P_1_ digestion of total RNA in indicated cells. Means ± s.e.m. from *n* = 8 samples each. **g** LC-MS analysis of tRNA^Phe^ nucleosides generated by purification of tRNA^Phe^ from total RNA of the indicated cells and nuclease P1 digestion. Means ± s.e.m. from *n* = 8 (Control + Empty) or *n* = 4 (Control + *DUS1L* and *DUS1L* KO + Empty) samples. **h** LC-MS analysis of RNase T_1_-digested fragments of tRNA^Tyr(GUA)^ purified from total RNA of the indicated cells. Extracted-ion chromatograms (XICs) of unmodified, single D-modified, and doubly D-modified 3-mer fragments (positions 16–18) with the indicated *m/z* are shown. NL, normalization level, n.d., not detectable. **b**–**d**, **f**, **g** ****P < 0.0001, ***P < 0.001, **P < 0.01, *P < 0.05, n.s., not significant by Brown–Forsythe and Welch ANOVA tests. Exact *P*-values are included in the Supplementary Data 1.

Human DUS1L is an ortholog of *S. cerevisiae* Dus1p (Fig. 1c). To assess the capacity of DUS1L to mediate formation of D16 and potentially also D17 in human cytoplasmic tRNAs, *DUS1L* was knocked out in two glioblastoma cell lines (LNZ308 and U87) using the CRISPR/Cas9 system. Two single guide RNAs (sgRNAs) were designed against *DUS1L* exon 3, upstream of a conserved cysteine residue whose counterpart in *E. coli* DusA is required for D formation^19^. After transduction of LNZ308 and U87 cells with lentiviruses encoding CRISPR/Cas9, single cells were cloned. We confirmed nucleotide deletions or insertions in the *DUS1L* gene, which caused frameshifts and generation of premature termination codons (Fig. 2a, Supplementary Fig. 1). Control cells were also generated by the same procedures using control sgRNAs that did not target the human genome. The *DUS1L* mRNA level was reduced in *DUS1L* KO cells (Fig. 2b), which suggests that *DUS1L* mRNAs bared premature termination codons and were thus degraded by the nonsense-mediated mRNA decay pathway^30^.

There was an approximately 30% decrease of D in total tRNA of *DUS1L* KO cells according to mass spectrometry of nucleosides obtained by degradation of gel-excised total cellular tRNAs (Fig. 2c). Given there are four human DUS paralogs, a 30% decrease of D upon *DUS1L* KO is reasonable. The decrease of D in *DUS1L* KO cells was confirmed to be due to loss of *DUS1L* by stably re-expressing sgRNA-resistant *DUS1L* in *DUS1L* KO cells (Fig. 2d, e, Supplementary Fig. 2), which recovered the D level in total RNA (Fig. 2f). *DUS1L* was also stably overexpressed in control cells (Fig. 2d–f, Supplementary Fig. 2), which we used to investigate the effect of *DUS1L* overexpression in later experiments. In addition, we attempted to overexpress catalytically dead mutant DUS1L as a negative control by mutating a cysteine residue whose counterpart in *E. coli* DusA is required for D formation^19^. However, upon expression of the DUS1L C107A mutant (equivalent to the *E. coli* DusA C114A mutant^19^), cell growth completely ceased (Supplementary Fig. 3), implying that this single point substitution has a dominant negative impact on cell growth, thus underscoring the importance of cysteine 107.

Various human cytoplasmic tRNAs, including tRNA^Phe^, tRNA^Tyr(GUA)^, tRNA^Ala(AGC)^, tRNA^Asn^, tRNA^His^, and tRNA^Val^, bear the D16 and/or D17 modifications^4^. To investigate whether or not DUS1L installs D16/D17 modifications into these tRNAs, cytoplasmic tRNA^Phe^, which has D at positions 16, 17, and 47^4^, was purified using an oligo DNA probe and subsequently subjected to mass spectrometric analysis to monitor the D level. The level of D was reduced in tRNA^Phe^ in *DUS1L* KO cells (Fig. 2g, red bar). Considering that D47 is modified by DUS3L^28^, this result strongly suggests that DUS1L is required for D16/D17 modification of tRNA^Phe^. To provide definitive evidence that DUS1L is required for D formation at tRNA positions 16 and 17, cytoplasmic tRNA^Tyr(GUA)^ was purified using a DNA probe, digested with G-specific RNase T_1_, and subjected to mass spectrometric analysis. tRNA^Tyr(GUA)^ fragments derived from control cells contained a 3-nt DDG fragment with both D16 and D17 modifications and lacked both the detectable unmodified UUG triplet as well as the single D-modified fragments (Fig. 2h). Thus, tRNA^Tyr(GUA)^ in control cells was fully D-modified at positions 16 and 17. On the contrary, *DUS1L* KO cells completely lacked both D16 and D17 modifications in tRNA^Tyr(GUA)^, and only the unmodified UUG fragment was detected. The loss of D16 and D17 modifications in *DUS1L* KO cells was rescued by stable re-expression of *DUS1L* (Fig. 2h). Therefore, *DUS1L* is required for both D16 and D17 modifications in cytoplasmic tRNA of human cells.

### In vitro reconstitution of D16 and D17 with recombinant DUS1L

**Fig. 3:**
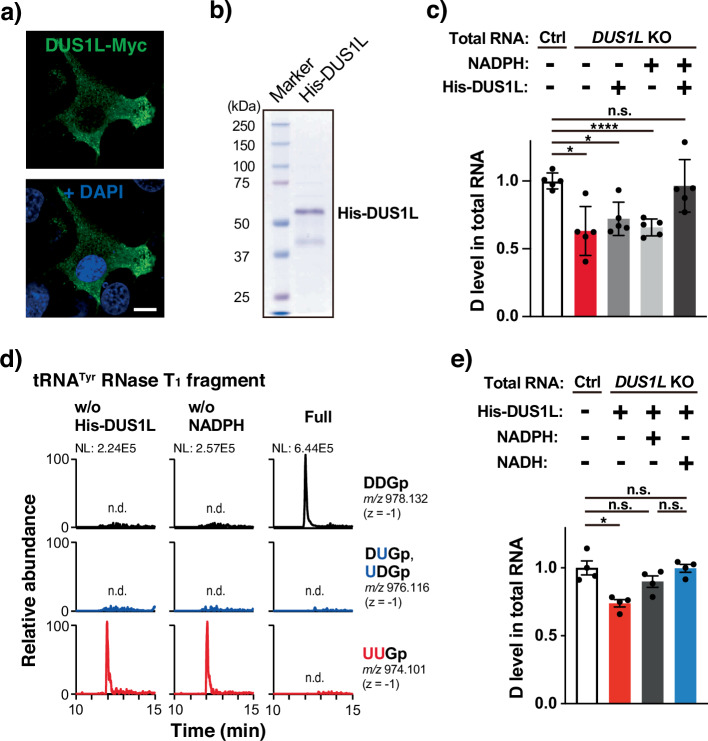
In vitro reconstitution of tRNA D16 and D17 modifications. **a** Subcellular localization of human DUS1L-Myc. U87 cells were transfected with a plasmid expressing DUS1L-Myc. Confocal microscopic images were obtained after immunostaining of Myc-tagged proteins (green) and DAPI staining (blue). Scale bar, 10 µm. **b** Purified His-DUS1L resolved by SDS-PAGE and stained with Coomassie Brilliant Blue. Protein size markers are indicated. **c** LC-MS analysis of total RNA nucleosides after incubating the reaction mixture with (+) or without (−) His-DUS1L or NADPH. Total RNA from *DUS1L* KO or control LNZ308 cells was used as the substrate RNA. The values are the relative levels to the mean D levels in total RNA of control cells, normalized by the uridine peak area of the same samples. Means ± s.e.m. from *n* = 5 samples each. **d** LC-MS analysis of RNase T_1_-digested fragments of tRNA^Tyr(GUA)^ purified from total RNA after incubating the reaction mixture with or without His-DUS1L or NADPH, using total RNA from *DUS1L* KO LNZ308 cells as the substrate RNA. XICs of unmodified, single D-modified, and doubly D-modified 3-mer fragments (positions 16–18) with the indicated *m/z* are shown. **e** LC-MS analysis of total RNA nucleosides after incubating the reaction mixture in the same way as in (c), with (+) or without (−) NADPH or NADH. Means ± s.e.m. from *n* = 4 samples each. ****P < 0.0001, *P < 0.05, n.s., not significant by the Brown–Forsythe and Welch ANOVA tests. Exact *P*-values are included in the Supplementary Data 1.

To investigate whether DUS1L is the enzyme that catalyzes tRNA D16 and D17 modifications, we performed in vitro reconstitution of D modifications using recombinant DUS1L protein. For the substrate RNA, rather than using an in vitro synthesized, unmodified tRNA transcript, we used total RNA of *DUS1L* KO cells. This is because confocal microscopic observation of C-terminally Myc-tagged human DUS1L (DUS1L-Myc) showed a predominantly cytoplasmic localization with a minor population localized to the nucleus (Fig. 3a), implying that DUS1L functions in the late steps of tRNA maturation. In addition, human DUS2L similarly localizes predominantly in the cytoplasm^27^, and yeast Dus2p requires prior installation of other tRNA modifications for its DUS activity^22^. We expressed hexahistidine-tagged DUS1L (His-DUS1L) in *E. coli* and purified His-DUS1L using Co^2+^-chelating beads (Fig. 3b). After the in vitro reconstitution reaction, the D level in total RNA increased only in the presence of both recombinant His-DUS1L and NADPH (Fig. 3c). Following the in vitro D reconstitution, tRNA^Tyr(GUA)^ was further purified from total RNA, digested with RNase T_1_, and subjected to mass spectrometric analysis. In the presence of both NADPH and His-DUS1L, uridines at positions 16 and 17 were fully D-modified in tRNA^Tyr(GUA)^ (Fig. 3d). Therefore, the human DUS1L enzyme catalyzes tRNA D16 and D17 modifications in vitro, without the need for additional partner proteins.

Bacterial DusA can use not only NADPH, but also NADH for the D modification^18^. In the case of eukaryotic DUS, although one study showed that recombinant human DUS2L can oxidize NADH^24^, no study likely has examined whether eukaryotic DUS can use NADH instead of NADPH for the D modification of tRNAs. To investigate this, an in vitro reconstitution experiment was performed in the same way as in Fig. 3c, except that NADH was used (Fig. 3e). As a result, NADH has been shown to be as effective as NADPH for the installation of D modification, producing similar D modification levels between the two, at least in terms of the end point of the reactions.

### DUS1L supports cell growth

**Fig. 4:**
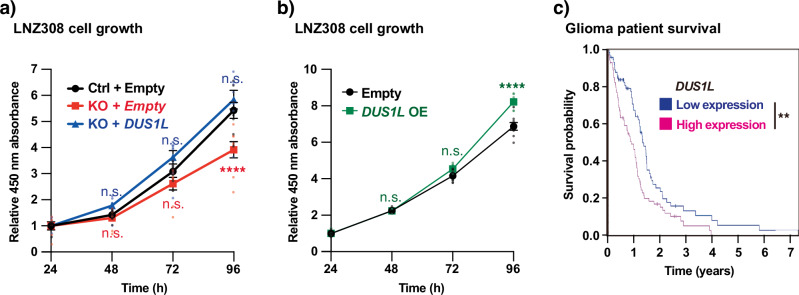
Effect of *DUS1L* expression on the growth speed of a glioblastoma cell line. Growth of *DUS1L* KO (**a**) and *DUS1L* OE (**b**) cells. The cell lines are the same as those used in Fig. 2. The cells were seeded at the same density at 0 h, and cell density was measured after 24, 48, 72, and 96 h using a Cell Counting Kit 8 and normalized by the cell density at 24 h. Means ± s.e.m. from *n* = 8 samples at each time point and cell type. ****P < 0.0001, n.s. not significant compared with control cells by a two-way ANOVA followed by a multiple comparison test. **c** Kaplan–Meier plot of glioma patients with higher or lower levels of tissue *DUS1L* mRNA expression and the *P*-value between the two groups (**P = 0.0024) acquired from the Human Protein Atlas database^31^. The cut-off FPKM value between high and low *DUS1L* expression was set to 7.47 in order to discern 76 patients with high *DUS1L* expression and 77 patients with low *DUS1L* expression.

To investigate if *DUS1L* is involved in cell proliferation, the proliferation rates of the cultured glioblastoma cell line LNZ308 were monitored. *DUS1L* KO reduced the growth rates to two-thirds of that of control cells, and this effect was fully reversed upon stable re-expression of CRISPR-resistant *DUS1L* (Fig. 4a). Moreover, *DUS1L* overexpression increased the growth rates of LNZ308 cells (Fig. 4b). Therefore, *DUS1L* supports growth of a glioblastoma cell line under a cultured condition.

Increased expression of tRNA modification enzymes is often associated with cancer progression and poor prognosis^5–7^. To investigate the possible association of *DUS1L* expression with cancer progression, the survival probabilities of glioma patients with higher and lower *DUS1L* mRNA expression levels were compared using data in the Human Protein Atlas database^31^. A Kaplan–Meier plot showed that increased expression of *DUS1L* was associated with poorer prognosis of glioma patients (Fig. 4c).

### *DUS1L* overexpression differentially affects the steady-state levels of the selected DUS1L-target tRNAs and increases the total cellular translation

**Fig. 5:**
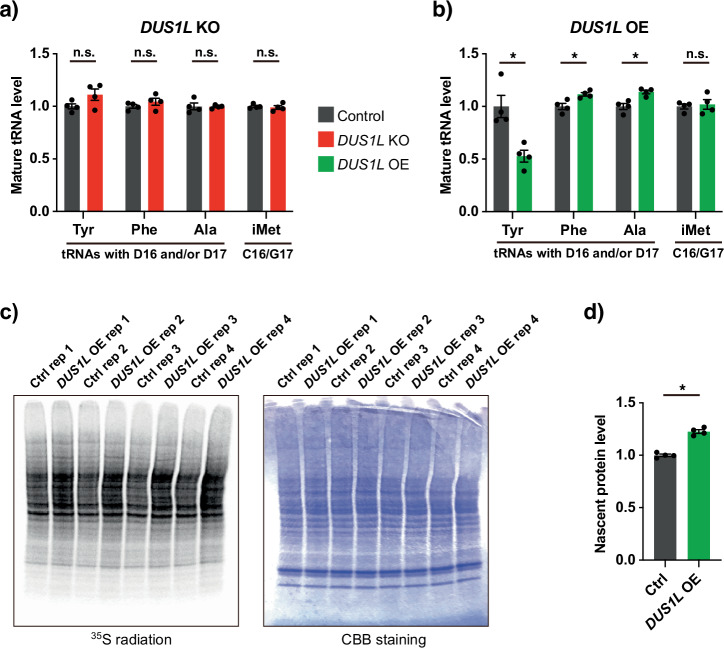
Effects of *DUS1L* overexpression on mature tRNA levels and total cellular translation. Mature tRNA levels in *DUS1L* KO (**a**) and *DUS1L* OE (**b**) cells. Northern blot analysis of tRNA^Tyr(GUA)^, tRNA^Phe(GAA)^, tRNA^Ala(AGC)^, and initiator tRNA^Met(CAU)^ was performed, followed by band quantification and normalization by 5.8S rRNA. The original images are shown in Supplementary Figs. 4 and 5. Means ± s.e.m. from *n* = 4 samples each. *P < 0.05 by the Mann–Whitney test. **c** Nascent cellular protein synthesis observed by ^35^S-methionine pulse labeling. A radiation image of the electrophoresed gels is shown on the left. Coomassie brilliant blue (CBB) staining of the same gel is shown on the right as a loading control. *n* = 4 samples each. **d** Quantification of the nascent protein levels in (**c**). Means ± s.e.m. from *n* = 4 samples each. *P < 0.05 by the Mann–Whitney test. Exact *P*-values are included in the Supplementary Data 1.

tRNA modifications are generally considered to support cell growth and functions by supporting protein synthesis^3,5,6^. To investigate the possible effect of DUS1L on tRNA levels and protein synthesis, we first monitored the steady-state levels of several D16- and/or D17-modified tRNAs^4^. Specifically, we performed northern blotting of D16/D17-bearing tRNA^Tyr(GUA)^, tRNA^Phe^, and tRNA^Ala(AGC)^, as well as D16/D17-lacking initiator methionine tRNA (tRNA^iMet^) as a control and found no changes in the steady-state levels of these tRNAs upon *DUS1L* KO (Fig. 5a, Supplementary Figs. 4a, 5). The overall cellular translation, monitored by ^35^S-methionine pulse labeling of nascent proteins, also did not show a clear difference in the overall translation level between *DUS1L* KO and control cells (Supplementary Fig. 4b).

By contrast, using the same stable *DUS1L-*overexpressing (OE) cells as in the experiments presented in Figs. 2 and 4, we found that *DUS1L* OE reduced tRNA^Tyr(GUA)^ by approximately half compared to the control cells (Fig. 5b). On the other hand, *DUS1L* overexpression slightly increased the steady-state level of D16/D17-modified tRNA^Phe^ (Fig. 5b, Supplementary Figs. 4c, 5), although it did not change the D level in tRNA^Phe^ (Fig. 2g). In addition, *DUS1L* overexpression also slightly increased the steady-state level of D17-modified^4^ tRNA^Ala(AGC)^, whereas the control D16/D17-lacking tRNA^iMet^ was unchanged (Fig. 5b, Supplementary Figs. 4c, 5). Interestingly, a ^35^S-methionine pulse-labeling experiment showed that *DUS1L* overexpression also increased overall cellular translation (Fig. 5c, d). We conclude that *DUS1L* overexpression differentially impacts the steady-state levels of several D16- and/or D17-modified tRNAs and increases the overall cellular translation. Since the levels of some tRNAs rise up, while those of tRNA^Tyr(GUA)^ drop down dramatically, the latter effect cannot be explained by a general increase in the amount of translational components, and its elucidation awaits further investigation.

### *DUS1L* overexpression suppresses 5′ and 3′ processing of the tRNA^Tyr(GUA)^ precursor and reduces the mature tRNA^Tyr(GUA)^ level

**Fig. 6:**
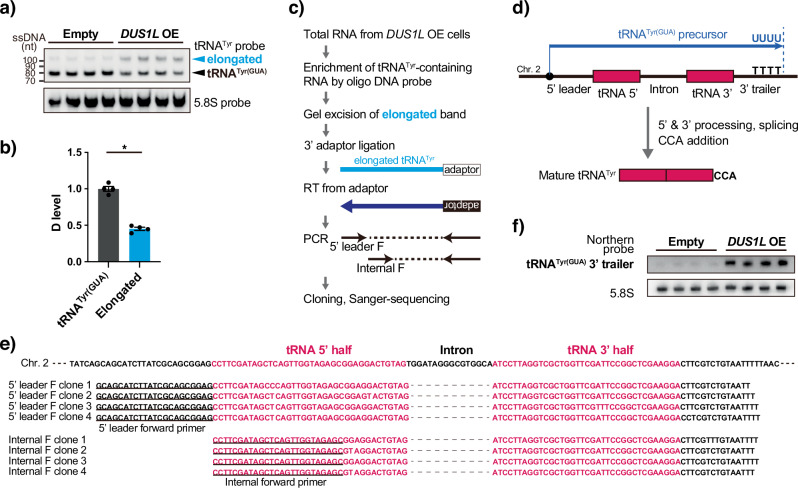
Effects of *DUS1L* overexpression on tRNA^Tyr(GUA)^ maturation. **a** Northern blot analysis of tRNA^Tyr(GUA)^ in *DUS1L* OE and control LNZ308 cells. Each lane was derived from different cell dishes. The lower band corresponds to mature tRNA^Tyr(GUA)^ and the upper band is elongated tRNA^Tyr(GUA)^. The positions of single-stranded DNA size markers are shown on the left. **b** Comparison of D levels in mature tRNA^Tyr(GUA)^ and the elongated band, which were enriched using an oligo DNA probe against tRNA^Tyr(GUA)^ and gel excision, followed by nuclease digestion and mass spectrometry analysis, normalized by cytidine levels. Means ± s.e.m. from *n* = 4 samples each. *P = 0.0286 by the Mann–Whitney test. **c** Outline of the method to identify the sequence of the elongated tRNA^Tyr(GUA)^. **d** Schematic of the gene structure, transcription, and post-transcriptional processing of human tRNA^Tyr(GUA)^. Pol III transcription terminates after producing the oligo(U) stretch^63^. The tRNA^Tyr(GUA)^ precursor contains the 5′ leader, intron, and 3′ trailer sequences, which are removed by RNase P, the tRNA splicing complex, and RNase Z, respectively, followed by the 3′ terminal CCA addition^64^. **e** Sanger sequencing results of the elongated tRNA^Tyr(GUA)^. The upper four clones were analyzed by PCR using a forward primer designed to target the 5′ leader (underlined sequences) and a reverse primer designed against the 3′ adapter. The lower four clones were analyzed by PCR using a forward primer designed to target the 5′ end inside the tRNA^Tyr(GUA)^ sequence (underlined sequences) and a reverse primer designed against the 3′ adapter. **f** Northern blot analysis using a probe designed against the tRNA^Tyr(GUA)^ trailer sequence. Each lane was derived from different cell dishes.

Northern blotting of tRNA^Tyr(GUA)^ (Fig. 5b) revealed that *DUS1L* OE cells accumulated a tRNA^Tyr^ band that was approximately 20–30 nt longer than wild type tRNA^Tyr^ (Fig. 6a, Supplementary Fig. 5). We hypothesized that this band might be a tRNA^Tyr^ precursor. To investigate this possibility, we first compared the D modification level of mature tRNA^Tyr(GUA)^ versus the elongated band. The tRNA^Tyr^-containing RNAs were enriched from total RNA of *DUS1L* OE cells using a biotinylated oligo DNA probe, and both forms were gel excised and subjected to nucleoside digestion followed by mass spectrometric analysis of the D level normalized to the cytidine level. We found that the elongated band contained about half the amount of D compared with mature tRNA^Tyr(GUA)^ (Fig. 6b). This result supported our precursor hypothesis and further suggested that DUS1L installs D16 and D17 in a step-wise manner, while stably interacting with this tRNA^Tyr(GUA)^. To further characterize the elongated tRNA^Tyr^ precursor, tRNA^Tyr^-containing RNAs were enriched from total RNA of *DUS1L* OE cells using a biotinylated oligo DNA probe, followed by gel excision of the elongated tRNA^Tyr^, 3′-adapter ligation, reverse transcription, PCR amplification, cloning, and Sanger sequencing (Fig. 6c). As expected, the elongated tRNA^Tyr^ was identified as the tRNA^Tyr(GUA)^ precursor retaining the 5′ leader and 3′ trailer sequences up to the oligo(U) transcriptional termination site but with the intron already spliced out (Fig. 6d, e). Accumulation of this tRNA^Tyr(GUA)^ precursor in *DUS1L* OE cells was confirmed by northern blotting using a probe against the trailer sequence of tRNA^Tyr(GUA)^ (Fig. 6f). Thus, *DUS1L* overexpression inhibits removal of the 5′ leader and 3′ trailer sequences of tRNA^Tyr(GUA)^ and reduces the mature tRNA^Tyr(GUA)^ level.

### *DUS1L* overexpression reduces translation of tyrosine codons and readthrough of UAA and UAG stop codons

**Fig. 7:**
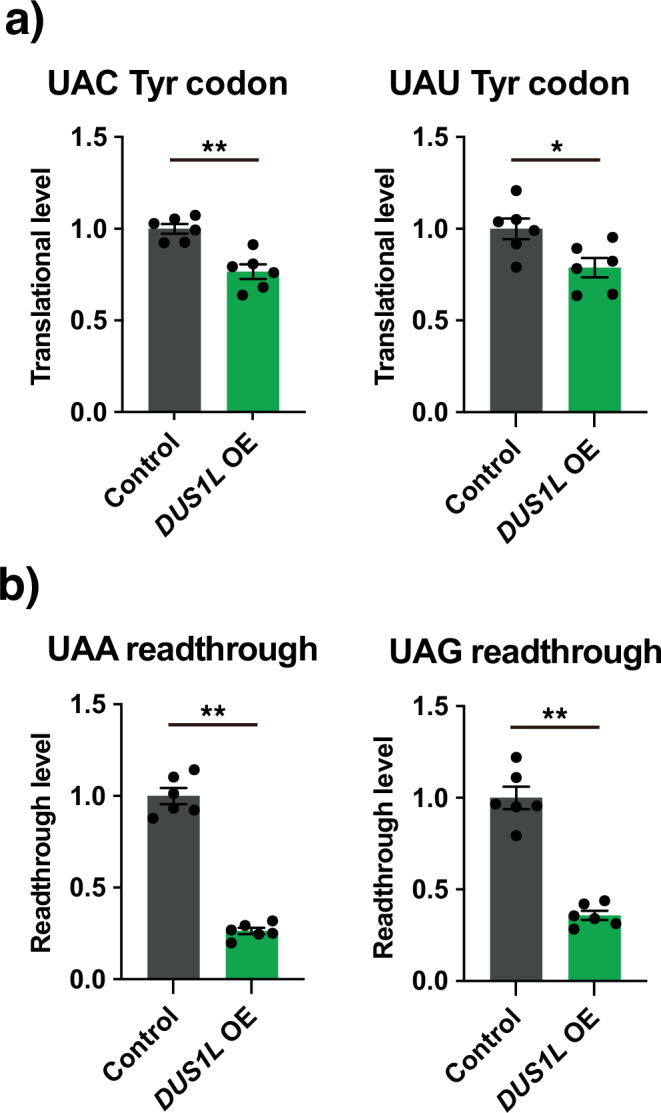
Effects of *DUS1L* overexpression on translation of tyrosine codons and translational readthrough of near-cognate stop codons. **a** Effects of *DUS1L* overexpression on translation of tyrosine codons. Cells were transfected with a codon translation reporter plasmid with a 5′ firefly luciferase sequence and a 3′ renilla luciferase sequence connected via five consecutive TAC, TAT, or random codons (control). Plasmid expression was normalized by dividing renilla luciferase luminescence by firefly luciferase luminescence. Possible renilla-to-firefly luciferase translational bias between control and *DUS1L* OE cells due to codons in luciferase genes was normalized using the random codon linker control. **b** Effects of *DUS1L* overexpression on translational readthrough of UAA and UAG stop codons. Cells were transfected with a stop codon readthrough reporter plasmid with a 5′ renilla luciferase sequence and a 3′ firefly luciferase sequence connected via a TAA codon (or its control TTA codon) or TAG codon (or its control TTG codon). Plasmid expression was normalized by dividing firefly luciferase luminescence by renilla luciferase luminescence. Possible firefly-to-renilla luciferase translational bias due to codons in luciferase genes was normalized using the corresponding TTA/TTG linker control. Means ± s.e.m. from *n* = 6 samples each. **P < 0.01, *P < 0.05 by the Mann–Whitney test. Exact *P*-values are included in the Supplementary Data 1.

To investigate if reduction of the tRNA^Tyr(GUA)^ levels upon *DUS1L* overexpression affects efficiency of translation of the tyrosine UAC and UAU codons, we performed codon translational reporter experiments using constructs with five consecutive codons of interest between the *firefly luciferase* and *renilla luciferase* genes. Translation of UAC and UAU codons was reduced by 23% and 21% in *DUS1L* OE cells, respectively (Fig. 7a). Thus, the *DUS1L* overexpression-mediated reduction of tRNA^Tyr(GUA)^ expectedly decreases translation efficiency of the UAC and UAU codons.

tRNA^Tyr(GUA)^, which decodes UAC and UAU codons, can sometimes mistranslate near-cognate UAA and UAG stop codons, by assigning a tyrosine to them, allowing so-called non-programmed stop codon readthrough^32^. To investigate whether the reduction of tRNA^Tyr(GUA)^ upon *DUS1L* overexpression affects stop codon readthrough, we performed stop codon readthrough reporter luciferase experiments. UAA and UAG readthrough was decreased in *DUS1L* OE cells (Fig. 7b). Therefore, the *DUS1L* overexpression-mediated reduction of tRNA^Tyr(GUA)^ decreases mistranslation of UAA and UAG codons.

## Discussion

In the present study, we identified DUS1L as the human enzyme responsible for D modifications at tRNA positions 16 and 17. Many tRNA modifications support mRNA translation and cell growth^3,5^. However, upon *DUS1L* KO, global nascent protein synthesis was not detectably reduced (Supplementary Fig. 4b), but cell growth was decreased (Fig. 4a). To understand this apparent discrepancy, the tRNA and codon translation levels may need to be monitored globally. Many enzymes that were previously regarded as tRNA modification enzymes were recently recognized to also serve as mRNA modification enzymes^33–35^. In the case of D, hundreds of D sites were recently found in yeast and human mRNAs^29,36^. Moreover, yeast Dus1–4p are responsible for D modifications of yeast mRNAs^29^. Our study may serve as a foundation to investigate the roles of D modifications not only in human tRNAs but also in human mRNAs. To investigate the possible roles of D modifications in mRNAs, performance of a combination of analyses such as ribosome profiling and transcriptome-wide mapping of D sites would be useful.

In *DUS1L* OE cells, the tRNA^Tyr(GUA)^ intron that extends from the anticodon loop of the tRNA^Tyr(GUA)^ precursor was spliced out, while 5′ and 3′ processing of the tRNA^Tyr(GUA)^ precursor was inhibited. RNase P is responsible for maturation of the 5′ end of precursor tRNAs. Human nuclear RNase P interacts with the T-loop, D-loop, and acceptor stem of precursor tRNAs^37,38^. By contrast, the tRNA splicing endonuclease interacts with intron-containing tRNAs by recognizing the anticodon stem and some regions of the D-arm and acceptor stem but without touching the tRNA “elbow”^39,40^. Thus, the small fraction of DUS1L that was observed in the nucleus (Fig. 3a) may have been bound to the tRNA^Tyr(GUA)^ precursor immediately after its transcription around the tRNA “elbow”, similar to other DUS enzymes that bind to this region^24,26^. Suppression of tRNA processing by a tRNA modification enzyme likely has not been reported previously. Thus, although it is in an overexpression context, this study may expand our knowledge of the functions of tRNA modification enzymes. It should be stressed here that tRNA 5′ and 3′ processing was likely not inhibited in other monitored D16/D17-containing tRNAs (tRNA^Phe^ and tRNA^Ala(AGC)^) (Supplementary Figs. 4c, 5). Currently, the cause for these differences is unclear; for example, it might be due to different DUS1L modes of binding to or affinity for different tRNA species.

As could have been anticipated, suppression of 5′ and 3′ processing of tRNA^Tyr(GUA)^ upon *DUS1L* OE was associated with reduction in the mature tRNA^Tyr(GUA)^ levels. Correctly processed 5′ and 3′ ends in the nucleus are recognized by the Exportin-t, which only exports such processed tRNAs to the cytoplasm^41,42^. Thus, upon *DUS1L* overexpression, suppression of 5′ and 3′ processing of precursor tRNA^Tyr(GUA)^ likely inhibited nuclear export of unprocessed tRNA^Tyr(GUA)^, allowing unprocessed tRNA^Tyr(GUA)^ to be targeted by the nuclear RNA degradation machinery.

We unexpectedly observed slight but significant increases of mature tRNA^Phe^ and tRNA^Ala(AGC)^ levels upon *DUS1L* overexpression (Fig. 5b, Supplementary Figs. 4c, 5). The increased amounts of several tRNAs might partially explain the increases of both cellular translation (Fig. 5c, d) and growth rates (Fig. 4b). However, this explanation must be taken with caution, because: (1) tRNA^Tyr(GUA)^ levels drop down dramatically and (2) there are other D16/D17-modified tRNAs^4^ with so far unknown impact of *DUS1L* overexpression on their steady-state levels. In yeast and Drosophila, overexpression of elongation factor 1 A, which binds to aminoacylated tRNAs, protects certain hypomodified tRNAs from degradation by the rapid tRNA decay pathway^43,44^. It is tempting to speculate that overexpressed DUS1L protein might similarly bind to and protect D16/D17-target tRNAs from degradation, but such a speculation demands future investigations.

Upon *DUS1L* overexpression, translation of UAC and UAU tyrosine codons and translational readthrough of near-cognate UAA and UAG stop codons were fittingly decreased (Fig. 7), which is likely attributable to a decreased level of mature tRNA^Tyr(GUA)^ (Fig. 5b). In addition, higher *DUS1L* expression was associated with poorer prognosis of glioma patients and *DUS1L* affected the growth rates of the selected cancer cell line (Fig. 4). UAA and UAG stop codon readthrough and tyrosine codon translation may affect the quality, quantity, and constituents of the proteome, and thus the cell growth. Further investigations are required to determine whether increased *DUS1L* expression in cancer tissues, which is likely lower than the expression level achieved in vector-mediated *DUS1L* OE cells (Fig. 2d), changes the proteome of cancer cells. To investigate this possibility in glioma patients, it is necessary to first investigate if glioma tissues with higher *DUS1L* expression contain more tRNA^Tyr(GUA)^ precursor, tRNA^Phe^, and tRNA^Ala(AGC)^ and less mature tRNA^Tyr(GUA)^. In addition, ribosome profiling would be useful to examine possible changes in sense codon dwelling times and stop codon readthroughs on UAR stops on translatome-wide level with a single nucleotide-level resolution.

In summary, we identified DUS1L as the human tRNA D16 and D17 modification enzyme, providing the foundation to study the roles of D modifications in physiology and pathophysiology.

## Methods

### Cell culture

The glioblastoma cell lines LNZ308 and U87 (gifts from Dr. Takahiro Yamamoto, Kumamoto University) were grown in Dulbecco’s modified Eagle’s medium (Thermo Fisher Scientific, 11995) supplemented with 10% fetal bovine serum (FBS) at 37 °C in a humidified atmosphere containing 5% CO_2_. Mycoplasma tests have not been performed. For cell growth analysis, cells were trypsinized and 1000 cells per 100 µL for each well were seeded in four 96-well plates and cultured in a CO_2_ incubator for 24, 48, 72, or 96 h. As blank controls, 100 µL of medium were transferred to four 96-well plates and put in a CO2 incubator for 24, 48, 72, or 96 h. The relative cell density was measured using a Cell Counting Kit 8 (Dojindo, CK04) according to the manufacturer’s protocol. Briefly, 10 µL of Cell Counting Kit 8 reagent was added to the wells containing cells or control medium and pipetted several times, followed by incubation in a 37 °C CO_2_ incubator for 1 h. Subsequently, the cell-containing 96-well plate and blank control 96-well plate were subjected to absorbance measurement at 450 nm using a Sunrise plate reader (TECAN). The absorbance in the blank medium well was subtracted as a background. The cell density was normalized by that at 24 h.

### Immunofluorescence

Immunofluorescence experiments were performed essentially as previously described^45,46^. *DUS1L* cDNA was amplified and a C-terminal Myc tag was added by reverse transcription and RT-PCR of total RNA from U87 cells using the primers listed in Supplementary Table 1. The amplified DNA was purified by agarose gel excision and cloned using a pcDNA3.4 TOPO Cloning Kit (Thermo Fisher Scientific, A14697) to generate the *DUS1L-Myc* expression vector (DUS1L-Myc/pcDNA3.4). U87 cells were grown on a 35 mm glass-bottom dish (Iwaki, 3911-035). The next day, the cells were transfected with 2.5 µg of DUS1L-Myc/pcDNA3.4 plasmid DNA using Lipofectamine 3000 (Thermo Fisher Scientific, L3000015). After 2 days, the cells were briefly washed with phosphate-buffered saline (PBS), fixed with 4% paraformaldehyde diluted in PBS (Wako, 10010) at room temperature for 10 min, washed thrice with PBS, and permeabilized with 0.2% Triton X-100 diluted in PBS at room temperature for 15 min. The cells were washed thrice with PBS containing 0.1% Tween (PBST), blocked with 1% bovine serum albumin diluted in PBST at room temperature for 1 h, and incubated with an anti-Myc antibody (MBL, M047-3, 1:500 dilution in PBST) at room temperature for 1.5 h. The cells were then washed thrice with PBST and incubated with an anti-mouse secondary antibody conjugated with Alexa Fluor 488 (Thermo Fisher Scientific, A21202, 1:500 dilution in PBST) at room temperature for 1 h. The cells were washed twice with PBST, stained with DAPI, and washed again with PBST. Finally, images were acquired using a FLUOVIEW FV3000 confocal microscope (Olympus).

### Generation of *DUS1L* KO and control cells

*DUS1L* KO cells were generated using the CRISPR/Cas9 system, as described previously^47,48^. Briefly, each set of sense and antisense oligonucleotides (two sets targeting *DUS1L* and two sets that did not target the human genome as negative controls, Supplementary Table 1) encoding a sgRNA were selected from the human GECKO v2 library^49^. Sense and antisense oligonucleotides were annealed and cloned into the BsmBI sites of the lentiCRISPR v2 blast plasmid (Addgene, 12260). Lentiviruses were generated by transfecting HEK293FT cells with the sgRNA sequence-containing lentiCRISPR v2 blast plasmid, psPAX2 (Addgene, 12260), and pMD2.G (Addgene, 12259). After 1 day, the lentivirus-containing medium was collected, filtered through a 0.45 µm Millex-HV filter (Millipore, SLGVR33RS), and added to LNZ308 or U87 cells for transduction of the generated lentiviruses. After blasticidin selection of transduced cells, single clones were acquired by limiting dilution.

### RNA extraction and RT-qPCR

LNZ308 or U87 cells in 10-cm dishes were briefly washed with PBS and lysed in 1 mL of TRI Reagent (MRC, TR118), and then total RNA was extracted according to the manufacturer’s protocol. RT-qPCR was performed as previously described^50^ using PrimeScript RT Master Mix (TAKARA, RR036A), Rotor Gene 2 (Qiagen), TB Green Premix Ex Taq II (TAKARA, RR820), and the primers listed in Supplementary Table 1.

### Stable re-expression of *DUS1L* in LNZ308 cells

*DUS1L-Myc* was PCR-amplified from the DUS1L-Myc/pcDNA3.4 plasmid using the primers listed in Supplementary Table 1 to add the CACC sequence at the 5′ terminus. To generate the DUS1L-Myc/pENTR plasmid, the PCR product was gel-excised and subcloned into the pENTR plasmid using a Directional TOPO Cloning Kit (Thermo Fisher Scientific, K240020SP). To re-express DUS1L-Myc in *DUS1L* KO2 cells (which stably expressed CRISPR/Cas9 with a sgRNA against the *DUS1L* coding sequence), the PAM-containing sequence CTGGCT (PAM underlined, encoding a leucine and an alanine) was synonymously mutated to CTAGCT in order to destroy the PAM sequence. The mutation was performing by PCR of the DUS1L-Myc/pENTR plasmid using the primers listed in Supplementary Table 1. Subsequently, the PCR product was transformed into *E. coli* to obtain the PAM-destroyed DUS1L-Myc/pENTR plasmid. To generate C107A mutant DUS1L-Myc/pENTR, WT DUS1L-Myc/pENTR was modified using the primers listed in Supplementary Table 1. WT or C107A mutant DUS1L-Myc was subcloned into pLX302 (Addgene, 25896) using LR Clonase II Enzyme Mix (Thermo Fisher Scientific, 11791020). HEK293FT cells were transfected with PAM-destroyed DUS1L-Myc/pLX302 (or empty pLX302 as the empty control), psPAX2 (Addgene, 12260), and pCMV-VSVG (Addgene, 8454) using Lipofectamine 3000 according to the manufacturer’s protocol. The medium was replaced with fresh medium approximately 12 h post-transfection. At 24 h after the medium change, medium containing *DUS1L-Myc*-encoding lentivirus particles (or empty control lentivirus particles) was collected, filtered, and added to Control 1 or *DUS1L* KO2 LNZ308 cells. After 4 days of culture in DMEM containing 10% FBS, transduced LNZ308 cells were selected by culture in medium containing 2 µg mL^−1^ puromycin for 3 days.

### Western blotting

Western blotting was performed as previously described^51,52^. Briefly, cells were trypsinized, washed with PBS, pelleted, lysed with lysis buffer (150 mM NaCl, 100 mM Tris-HCl pH 8, 0.5% NP-40, and a protease inhibitor cocktail (Roche, 05056489001)), and sonicated for 10 s. The protein concentration was determined using a Pierce BCA Protein Assay Kit (Thermo Fisher Scientific, 23225). Samples were electrophoresed on a sodium dodecyl sulfate (SDS) polyacrylamide gel and transferred to an Immobilon-P membrane (Merck Millipore, IPVH00010). The membrane was blocked with 5% skim milk prepared in TBST buffer (150 mM NaCl, 25 mM Tris-HCl pH 7.4, 2.7 mM KCl, and 0.05% Tween-20) and probed using a primary antibody against DUS1L (Santa Cruz, sc-393586, 1:100) or actin (MBL, M177-3, 1:5000) diluted in 5% skim milk prepared in TBST buffer at 4 °C overnight. The membrane was washed in TBST, probed using a secondary antibody (Dako, 0477, 1:4000) at room temperature for 1 h, and washed in TBST. Signals were detected using ECL Prime Western Blotting Detection Reagent (Cytiva, RPN2236) and a ChemiDoc imager (Bio-Rad).

### RNA nucleoside mass spectrometry

RNA nucleoside mass spectrometry was performed as previously described^53,54^. Briefly, 20 µL of solution containing total RNA (3 µg for LCMS-8050 or 1 µg for LCMS-8060), 20 mM Hepes-KOH (pH 7.6), 1.6 U of nuclease P_1_ (Fujifilm, 145-08221), and 0.2 U of bacterial alkaline phosphatase (Takara, 2120 A) was incubated at 37 °C for 3 h. Subsequently, 3 µL of the nucleoside solution was injected into a LCMS-8050 or LCMS-8060 system (Shimadzu). The nucleosides were first separated by an Inertsil ODS-3 column (GL Science, 5020-84655) using a mobile phase that continuously changed from 100% solution A (5 mM ammonium acetate in water, pH 5.3) to 100% solution B (60% acetonitrile in water) over 17 min at a flow rate of 0.4 mL min^−1^, followed by electrospray ionization and triple quadrupole mass spectrometry in multiple reaction monitoring mode.

### Isolation of tRNA^Phe^ and tRNA^Tyr(GUA)^ from total RNA

tRNA^Phe^ and tRNA^Tyr(GUA)^ were isolated from total RNA as previously described^47^. Briefly, 2 nmol of the 3′ biotinylated DNA probe (Supplementary Table 1) was bound to streptavidin beads (GE Healthcare, 17511301) at room temperature in binding buffer (100 mM NaCl, 10 mM Hepes-KOH pH 7.6, and 5 mM EDTA) for 1 h, followed by washing with binding buffer and hybridization buffer (1200 mM NaCl, 30 mM Hepes-KOH pH 7.6, and 7.5 mM EDTA). The probe-bound beads were mixed with 100 µg of total RNA in hybridization buffer and incubated at 65 °C for 1 h with occasional agitation. The beads were washed ten times with wash buffer (600 mM NaCl, 30 mM Hepes-KOH pH 7.6, and 7.5 mM EDTA) at 65 °C and eluted with elution buffer (20 mM NaCl, 0.5 mM Hepes-KOH pH 7.6, and 0.25 mM EDTA) at 65 °C. Subsequently, the eluate was subjected to 7 M urea/Tris-borate-EDTA (TBE)/10% polyacrylamide gel electrophoresis (PAGE), followed by SYBR Gold staining and gel excision of the tRNA band.

### RNase T_1_ fragment mass spectrometry

Mass spectrometry of tRNA fragments digested by RNase T_1_ was performed essentially as previously described^55^. The isolated tRNA^Tyr(GUA)^ (approximately 50 ng) was digested in 10 µL of reaction mixture containing 20 mM NH_4_OAc (pH 5.3) and 2 U µL^−1^ RNase T_1_ at 37 °C for 30 min and then mixed with a 1/10 volume of 0.1 M triethylamine-acetate (pH 7.0). The digests were subjected to capillary liquid chromatography (LC)/nano-electron spray ionization mass spectrometry (ESI-MS) using a DiNa splitless nano HPLC system (Techno Alpha) connected to a Q Exactive Hybrid Quadrupole-Orbitrap mass spectrometer (Thermo Fisher Scientific). The digests were loaded on a trap column (L-column2 ODS, 5 μm particle size, 0.3 × 5 mm; Chemical Evaluation and Research Institute) and separated on a HiQ sil C18W-3 nanospray column (C18, 3 μm particle size, 0.1 × 100 mm; Techno Alpha) with a linear gradient of 2.5–50% methanol containing 0.4 M 1,1,1,3,3,3-hexafluoro-2-propanol (pH 7.0, adjusted with triethylamine) over 35 min at a flow rate of 300 nL min^−1^. The scan range was *m/z* 600–2000.

### Expression and purification of recombinant His-DUS1L protein

The human *DUS1L* coding sequence with codons optimized for *E. coli* was synthesized by Azenta Life Sciences. The sequence was amplified using the primers listed in Supplementary Table 1 to add 5′ NheI and 3′ BamHI recognition sequences. The PCR amplicon was cloned into the pET28a (+) vector (Novagen) to obtain the expression vector pET-DUS1L, which produced N-terminal hexahistidine tag-fused DUS1L (His-DUS1L). One Shot BL21 Star DE3 cells (Thermo Fisher Scientific, C601003) were transformed with pET-DUS1L and cultured in LB media containing 50 µg mL^−1^ kanamycin. When the bacteria reached an OD of 0.5, protein expression was induced by addition of 100 µM isopropyl β-D-thiogalactopyranoside, and the cells were grown for 3 h at 37 °C. Cells were harvested, and the pellet (2 g) was suspended in 40 mL of lysis buffer (50 mM Hepes-KOH pH 7.6, 250 mM KCl, 1 mM 2-mercaptoethanol (2-ME), 1× protease inhibitor cocktail (Roche, 05056489001), 200 µg mL^−1^ lysozyme (Fujifilm, 129-06723), and 1/1000 volume DNaseI (Roche, 4716728001)). The lysate was sonicated on ice using a Branson 250 Sonifier (power 6, total of 3 min) and cleared by centrifugation at 12,000 × *g* for 20 min. Thereafter, 1 mL of 50% slurry TALON Metal Affinity Resin (TAKARA, 635502) was washed with equilibration buffer (lysis buffer without lysozyme and DNaseI), mixed with the *E. coli* lysate, and rotated at 1 h at 4 °C. The beads were cleared and washed with 10 mL of the following buffers: twice with equilibration buffer, four times with wash buffer 1 (20 mM Hepes-KOH, pH 7.6, 500 mM NaCl, 0.5% Triton X-100, and 1 mM 2-ME), four times with equilibration buffer, and once with wash buffer 2 (50 mM Hepes-KOH pH 7.6, 250 mM KCl, 1 mM 2-ME, and 10 mM imidazole pH 7.6). Ten elution fractions were collected each using 600 µL of elution buffer (50 mM Hepes-KOH pH 7.6, 250 mM KCl, 1 mM 2-ME, and 150 mM imidazole pH 7.6). Fractions 3–6 were collected, dialyzed overnight in dialysis buffer (50 mM Hepes-KOH pH 7.6, 150 mM KCl, and 1 mM dithiothreitol (DTT)), aliquoted, snap-frozen in liquid nitrogen, and stored at −80 °C until the in vitro reconstitution experiment.

### In vitro reconstitution of D using recombinant His-DUS1L

In vitro D reconstitution was performed essentially as previously described for DUS2L^24^. The in vitro reconstitution reaction mixture (50 µL) contained 50 mM Tris-HCl (pH 7.5), 100 mM CH_3_COONa, 10 mM MgCl_2_, 2 mM DTT, 1 mM NADPH (or 1 mM NADH), 15% glycerol, 1 µM His-DUS1L, and 25 µg of total RNA from *DUS1L* KO LNZ308 cells. The reaction mixture was incubated at 30 °C for 2 h. The reaction was terminated by adding TRI Reagent, and RNA was recovered according to the manufacturer’s protocol.

### Northern blotting

Northern blotting was performed as previously described^56^. Total RNA (1.5 µg) was first separated by 7 M urea/TBE/10% PAGE. The gel was stained with SYBR Gold to check RNA quality and then transferred to a nylon membrane (Merck Millipore, INYC00010) in 1× TBE using a wet transfer blotting system (Bio-Rad) on ice at 50 V for 80 min. Membranes were dried and crosslinked with ultraviolet light at 1200 × 100 µJ cm^−2^ using HL-2000 Hybrilinker (Funakoshi) and incubated in prehybridization buffer (6× saline sodium citrate (SSC), 0.1% SDS, and 1× Denhardt’s solution) at 42 °C for 1 h. The membranes were then hybridized with digoxigenin (DIG)-labeled probe DNA (Roche) in hybridization buffer (900 mM NaCl, 90 mM Tris-HCl pH 8, 6 mM EDTA, and 0.3% SDS) overnight at 50 °C. The probes for tRNA^Phe^, tRNA^iMet^, and 5.8S rRNA were the same as those used in a previous study^57^. The probes for tRNA^Tyr(GUA)^ and tRNA^Ala(AGC)^ were designed for this study. The sequences of northern blot probes are listed in Supplementary Table 1. The membranes were washed with 1× SSC, blocked using a DIG Wash and Block Buffer Set (Roche, 11585762001), and probed with anti-DIG alkaline phosphatase Fab fragments (Roche, 11093274910) and CDP-Star (Roche, 12041677001). Images were acquired using a ChemiDoc imager (Bio-Rad). The band intensities were quantified using ImageJ software^58^.

### ^35^S-methionine labeling of nascent proteins

Pulse labeling of nascent proteins was performed essentially as previously described^47,48^. One day before radiolabeling, 6 × 10^5^ cells were seeded into 6-cm dishes. The next day, the cells were briefly washed using 37 °C DMEM without Met, Cys, and Gln (Thermo Fisher Scientific, 21013024). A total of 3 mL of 37 °C preincubation medium (DMEM without Met, Cys, or Gln, supplemented with 2% FBS, 2 mM Gln, and 0.2 mM Cys) was added to the cells, and the cells were incubated in a CO_2_ incubator at 37 °C for 15 min. The medium was then exchanged with 1.25 mL of incubation medium (1.25 mL of preincubation medium containing 8.88 MBq of ^35^S-labeled methionine [PerkinElmer]), and the cells were incubated in a CO_2_ incubator at 37 °C for 30 min. Subsequently, the medium was removed and the cells were washed with PBS and collected using trypsin and DMEM containing 10% FBS. Cells were lysed and the protein concentration was measured using a BCA Protein Assay Kit (Thermo Fisher Scientific, 23225). Then, 15 µg of total protein were run on a Tricine PAGE gel (NOVEX, EC66252BOX). The gel was stained using Coomassie Brilliant Blue staining solution (Bio-Rad, 1610436), dried on a gel dryer, and photographed. A radiation image was acquired using an imaging plate and Typhoon FLA 7000 imager (GE Healthcare).

### Identification of the tRNA^Tyr^-containing band that accumulated upon *DUS1L* overexpression

tRNA^Tyr^-containing RNAs were isolated from total RNA of *DUS1L* OE cells using a biotinylated complementary DNA probe as described above. The tRNA^Tyr^-containing RNA was subjected to urea-PAGE, and the gel was stained with SYBR Gold. The band situated approximately 20–30 nt above the tRNA^Tyr^ band was gel-excised. The elongated tRNA^Tyr^ was demethylated using *E. coli*-derived AlkB (Addgene, 79050) to enable reverse transcription^59^. In brief, the RNA was incubated in 45 mM Tris-HCl (pH 8), 0.9 mM α-ketoglutaric acid, 1.8 mM ascorbic acid, 67 µM (NH_4_)_2_Fe(SO_4_)_2_, and 2.5 µM AlkB at 37 °C for 2 h. Demethylation was confirmed by simultaneously performing yeast tRNA demethylation and RNA nucleoside mass spectrometry as a positive control. The RNA and Adenylated Linker A (BIOO) were incubated at 60 °C for 3 min for denaturation and then ligated using T4 RNA ligase 2 truncated (NEB, M0242), according to the manufacturer’s protocol. The 3′ adapter-ligated tRNA was urea gel-excised and reverse-transcribed using ReverTra Ace (Toyobo, TRT-101) according to the manufacturer’s protocol and a primer listed in Supplementary Table 1. cDNA was gel-excised and PCR-amplified using a forward primer corresponding to the 5′ leader or tRNA internal 5′ region and a reverse primer complementary to the 3′ adapter (Supplementary Table 1). The PCR products were cloned using a TA Cloning Kit (Thermo Fisher Scientific, A14697), followed by Sanger sequencing of the clones.

### Luciferase reporter experiments

The translation reporter was constructed using a plasmid containing dual CMV promoters (PSF-CMV-CMV-SBF1-FLUC, Sigma-Aldrich, OGS608). cDNA encoding the *Renilla luciferase* gene was cloned into the XhoI and BamHI sites. Subsequently, oligonucleotides containing five consecutive TAC or TAT tyrosine codons (or five random codons for a control plasmid) were cloned at the 5′ end of the *Renilla luciferase* gene by insertion into NcoI and XhoI sites.

One day before plasmid transfection, 9 × 10^3^ LNZ308 cells were transferred to a 96-well plate. The next day, the cells were transfected with 10 ng of the codon reporter or control plasmid using Lipofectamine 3000. At 24 h post-transfection, the cells were lysed and luminescence was measured using a Dual-luciferase Reporter Assay System (Promega, E1910), a specialized 96-well plate (Greiner, 675075), and a Centro XS3 LB960 luminometer (Bethold Technologies). Differences of luminescence due to the transfected plasmid level were normalized using the internal firefly luciferase. Possible biases of the renilla-to-firefly luminescence ratio due to codons in the two luciferases were normalized using the control plasmid with random codons between the *renilla luciferas*e and *firefly luciferase* genes.

UAA and UAG readthrough reporter plasmids from previous studies^32,60^ were used. A UAA or UAG readthrough reporter plasmid expresses a transcript with a *renilla luciferas*e coding sequence at the 5′ side connected via a UAA or UAG codon to a *firefly luciferas*e coding sequence at the 3′ side. In addition, we generated corresponding control plasmids that expressed the *renilla*-*firefly* transcript with a UUA or UUG (leucine) codon linker instead of the UAA or UAG codon linker by performing PCR of the UAA or UAG codon reporter plasmids using the primers listed in Supplementary Table 1. The control plasmids were used to normalize possible biases of the renilla-to-firefly luminescence ratio due to codons in the two luciferases. Plasmid transfection and luciferase measurements were performed in the same way as described for codon translation measurement, except that 240 ng of plasmid was used for transfection.

### Statistics and reproducibility

Each n represents a distinct sample derived from cells grown in separate dishes or separate in vitro reconstitution reaction. All numerical data were analyzed by GraphPad Prism 9 or 10 software. No data were excluded. Blinding was not performed due to time and personnel constraints. To assess differences between two groups, the Mann–Whitney test was used. A two-tailed *P* value less than 0.05 was considered significant. To assess differences between more than two groups, an analysis of variance (ANOVA) or its derivatives was used. Data are presented as means ± standard error of means (s.e.m.). Exact *P*-values are included in the Supplementary Data 1.

### Reporting summary

Further information on research design is available in the Nature Portfolio Reporting Summary linked to this article.

## Supplementary information

Supplementary Information

Description of Additional Supplementary Materials

Dataset 1

Reporting Summary

## Data Availability

The source data underlying the graphs are available online. All data and cell resources presented in this study are available upon reasonable request.
