# Supplementary material for: Human DUS1L catalyzes dihydrouridine modification at tRNA positions 16/17, and DUS1L overexpression perturbs translation
Source: Commun Biol. 2024 Oct 2;7:1238. doi: 10.1038/s42003-024-06942-8 (PMC11445529; doi:10.1038/s42003-024-06942-8)
Supplement: Supplementary file 1 — Supplementary Information [file 42003_2024_6942_MOESM1_ESM.pdf]

Supplemental Information for

Human DUS1L catalyzes dihydrouridine modification at tRNA positions 16/17, and *DUS1L* overexpression perturbs translation

Jin Matsuura, Shinichiro Akichika, Fan-Yan Wei, Tsutomu Suzuki, Takahiro Yamamoto, Yuka Watanabe, Leoš Shivaya Valášek, Akitake Mukasa, and Kazuhito Tomizawa*, and Takeshi Chujo*

*Corresponding authors. Email:
tchujo@kumamoto-u.ac.jp (T.C.), tomikt@kumamoto-u.ac.jp (K.T.)

This file includes:

Supplementary Figures 1 – 5.

Supplementary Table 1.

Supplementary Figure 1. Confirmation of mutated *DUS1L* sequences in the generated cell lines

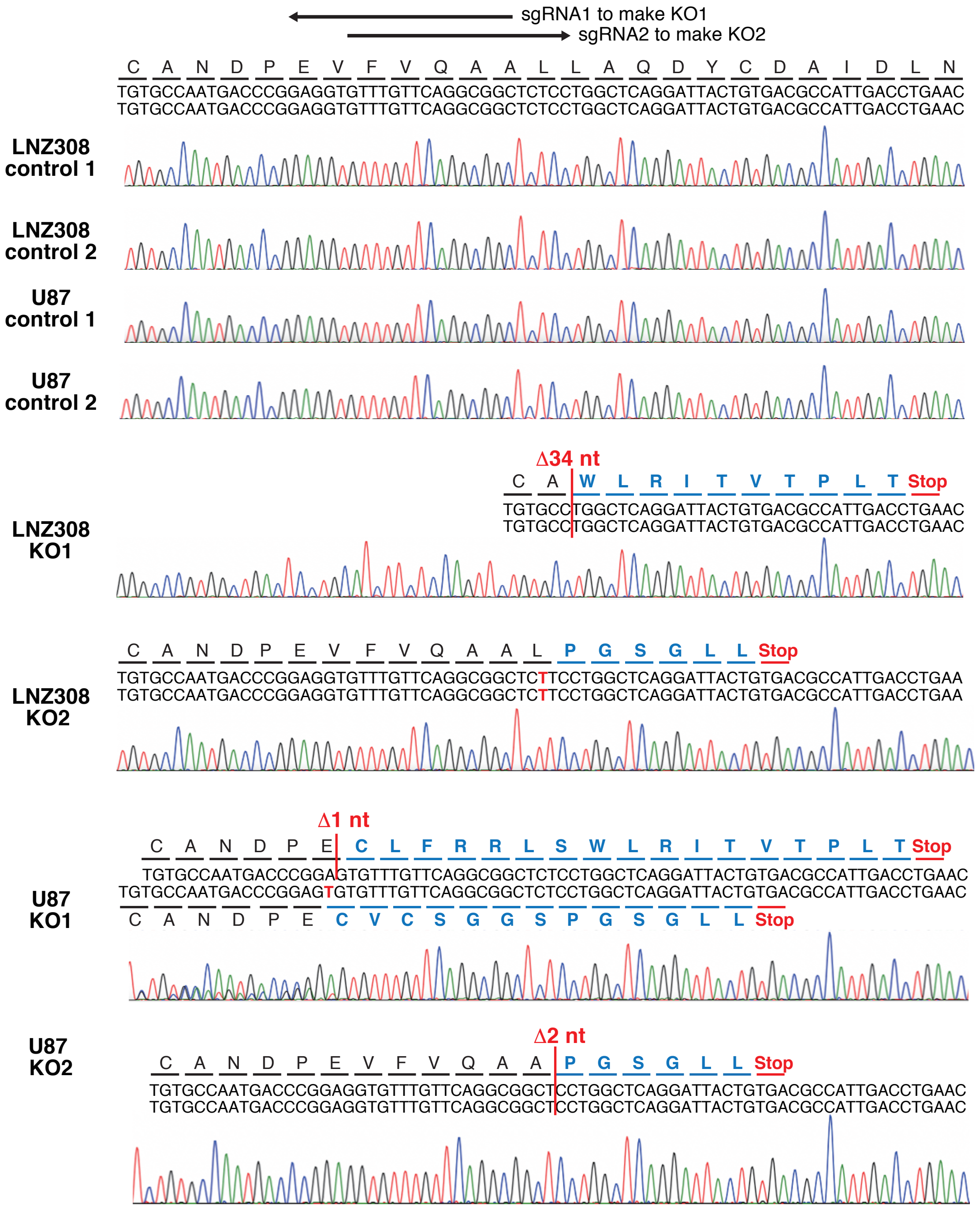

Supplementary Figure 1. Confirmation of mutated *DUS1L* sequences in the generated cell lines. The DNA regions near the sgRNA-targeted sequences were PCR-amplified and Sanger-sequenced. The sequencing primer was designed to target the reverse strand, and the sequences were read in the right-to-left direction. Reverse-complementary electropherograms were depicted using ApE software. Insertions, deletions, and premature termination codons are depicted in red letters. Amino acids translated from frameshifted codons are depicted in blue letters.

Supplementary Figure 2. Uncropped western blot images related to Figure 2e

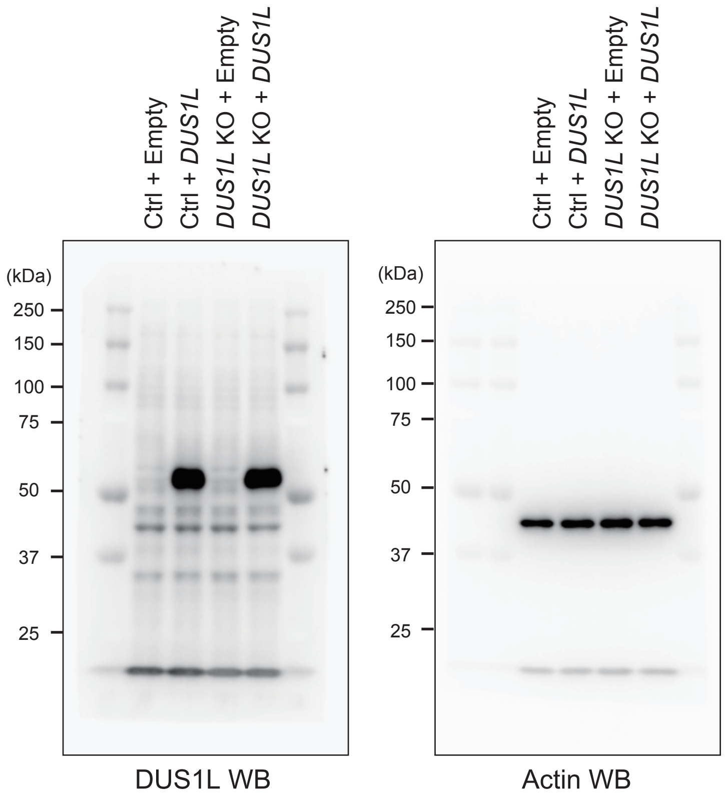

Supplementary Figure 3. Complete growth arrest of cells overexpressing C107A mutant DUS1L

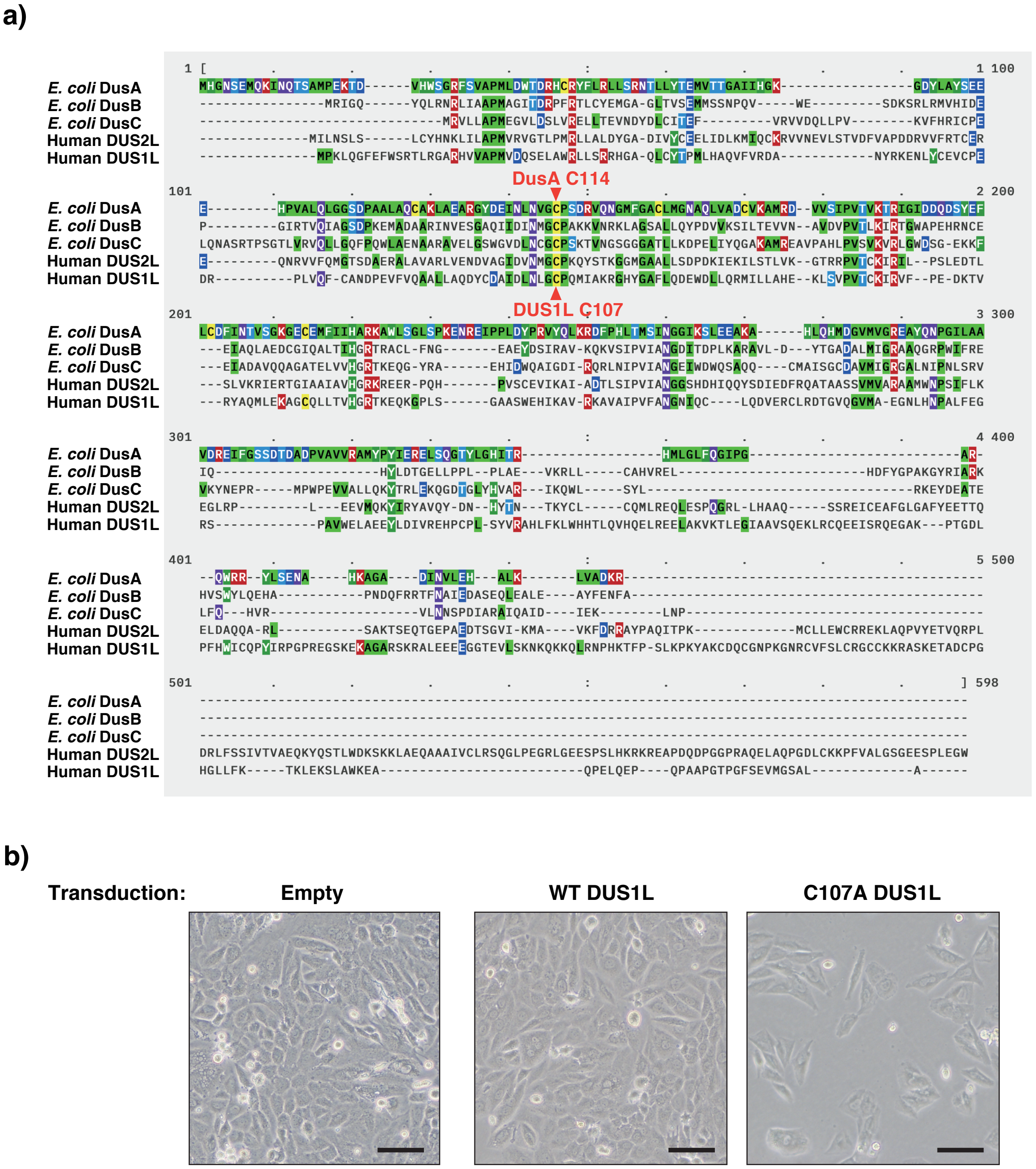

Supplementary Figure 3. Complete growth arrest of cells overexpressing C107A mutant DUS1L.
a Sequence alignment of *E. coli* DusA, *E. coli* DusB, *E. coli* DusC, human DUS2L, and human DUS1L. The protein sequences are the same as those used for phylogenetic analysis in Figure 1C. The sequences were subjected to multiple alignment by Clustal Omega and visualized with MView. Mutation of the conserved cysteine residue in *E. coli* DusA (marked by an arrowhead) to alanine disrupts dihydrouridylation activity (19). **b** Microscopic images of cells transduced with the empty vector, WT DUS1L expression vector, or C107A mutant DUS1L expression vector. C107A mutant DUS1L corresponds to the C114A mutant of *E. coli* DusA. Transduction of the C107A DUS1L expression vector completely halted cell growth. The images are of control cells at 6 days after transduction of the indicated lentiviral vectors. Scale bar, 100 μm.

Supplementary Figure 4. Levels of tRNAs and translation in *DUS1L* KO and *DUS1L* OE cells.

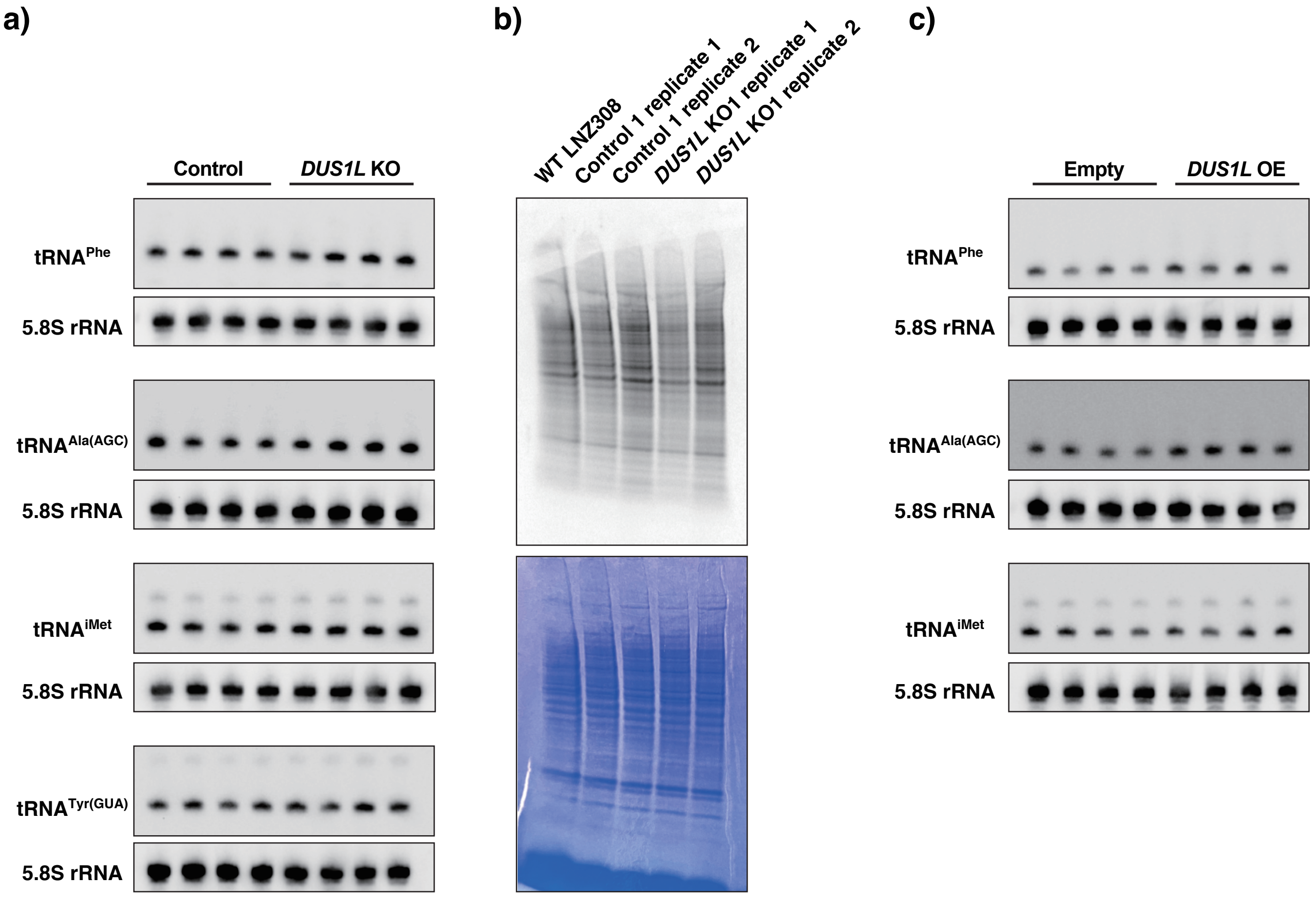

Supplementary Figure 4. Levels of tRNAs and translation in *DUS1L* KO and *DUS1L* OE cells.
a Northern blot images of $tRNA^{Phe}$, $tRNA^{Ala(AGC)}$, $tRNA^{iMet}$, $tRNA^{Tyr(GUA)}$, and loading control 5.8S rRNA. The band intensities were quantified in Figure 5a. Uncropped images are shown in Supplementary Figure 5. **b** Nascent cellular protein synthesis observed by ^{35}S-methionine pulse-labeling. A radiation image of the electrophoresed gel is shown at the top. Coomassie Brilliant Blue staining of the same gel as a loading control is shown at the bottom. **c** Northern blot images of $tRNA^{Phe}$, $tRNA^{Ala(AGC)}$, $tRNA^{iMet}$, and loading control 5.8S rRNA. The band intensities were quantified in Figure 5b. A northern blot image of $tRNA^{Tyr(GUA)}$ is shown in Figure 6a. Uncropped images are shown in Supplementary Figure 5.

.

Supplementary Figure 5. Uncropped northern blot images related to Figures 5 and 6

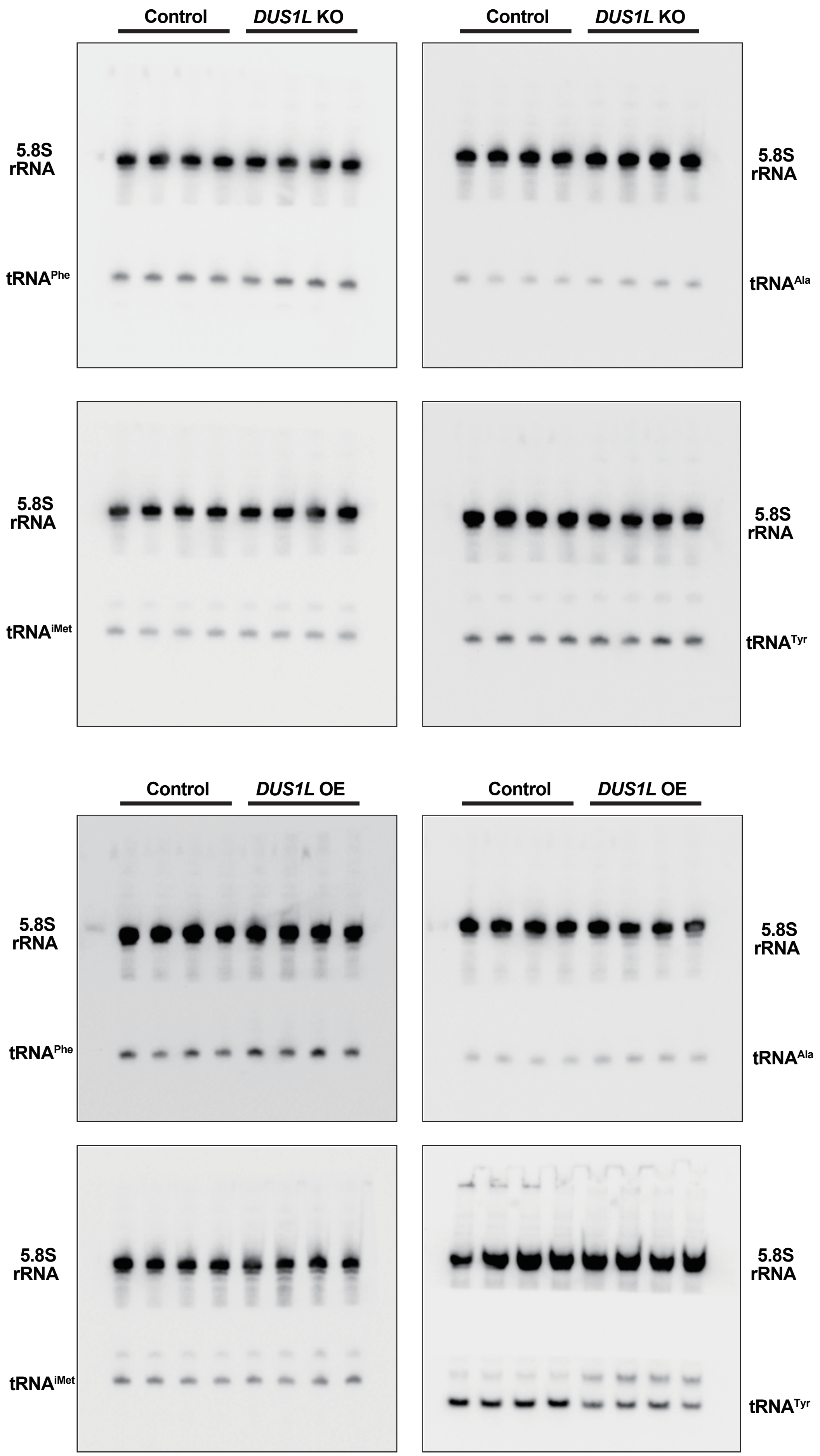

Supplementary Figure 5. Uncropped and unedited northern blot images related to Figures 5 and 6.
After electroblotting from SYBR Gold-stained gel, membranes were cut in half on SYBR Gold-stained 5S rRNA; lower half of the membrane was probed for tRNA and upper half was probed for 5.8S rRNA as a loading control. The corresponding tRNA membrane and 5.8S rRNA membrane were imaged together.

Supplementary Table 1. Oligo DNAs used in this study.

Name	Sequence
DUS1L-Myc ORF PCR forward	ATGCCAAAGCTGCAGGGC
DUS1L-Myc ORF PCR reverse	TCAAAGATCTTCTTCGCTAATAAGTTTTTGTTCTCCTGAGGCCA GGGCACTGC
Control sg1 top	CACCGACGGAGGCTAAGCGTCGCAA
Control sg1 bottom	AAACTTGCGACGCTTAGCCTCCGTC
Control sg2 top	CACCGCGCTTCCGCGGCCCGTTCAA
Control sg2 bottom	AAACTTGAACGGGCCGCGGAAGCGC
DUS1L sg1 top	CACCGGCCGCCTGAACAAACACCTC
DUS1L sg1 bottom	AAACGAGGTGTTTGTTCAGGCGGCC
DUS1L sg2 top	CACCGGTTTGTTCAGGCGGCTCTCC
DUS1L sg2 bottom	AAACGGAGAGCCGCCTGAACAAACC
Around CRISPR-target PCR forward	TGAGGGAAGAGAGAGCCAGT
Around CRISPR-target PCR reverse	TCCCGGCTTTGTCATATTGT
DUS1L qPCR forward	TTACTGTGACGCCATTGACC
DUS1L qPCR reverse	CTTTGGAGCAGGTCCCACT
GAPDH qPCR forward	GGGAAGCTTGTCATCAATGG
GAPDH qPCR reverse	TGGACTCCACGACGTACTCA
DUS1L-Myc ORF D-TOPO PCR forward	CACCATGCCAAAGCTGCAGGGC
DUS1L-Myc ORF PCR reverse	TCAAAGATCTTCTTCGCTAATAAGTTTTTGTTCTC
PAM mutant *DUS1L* PCR forward	TCAGGCGGCACTCCTAGCTCAGGATTACTG
PAM mutant *DUS1L* PCR reverse	ACAAACACCTCCGGGTCATTGGCACAGAAC
C107A mutation PCR forward	ACCTGAACTTGGGCGCCCCACAGATGATAG
C107A mutation PCR reverse	CTATCATCTGTGGGGCGCCCAAGTTCAGGT
DUS1L subcloning to pET28a forward	GCCACTAGTATGCCGAAACTCCAAGGCTTC
DUS1L subcloning to pET28a reverse	TCCTGAGAATTCTTACGCGAGGGCGCTG
tRNA Phe purification probe	CCTTTAGATCTTCAGTCTAACGCTCTCCCAACTGAGCTA/3Bio/
tRNA Tyr(GUA) purification probe	CTTCGAGCCGGAATCGAACCAGCGACCTAA/3Bio/
3' adaptor	5’rApp/CTGTAGGCACCATCAAT/ddC3’
3’ adaptor RT, PCR reverse	GTCATATGCGATTGATGGTGCCTACAG
$tRNA^{Tyr}$ internal PCR forward	CCTTCGATAGCTCAGTTGGTAGAGC
$tRNA^{Tyr}$ leader PCR forward	GCAGCATCTTATCGCAGCGGAG
tRNA Tyr northern probe	CTTCGAGCCGGAATCGAACCAGCGACCTAA
tRNA Phe northern probe	CGAAACCCGGGATCGAACCAGGGACCTTTA
tRNA Ala(AGC) northern probe	TCGTGCATGCCAAGCACGCGCTCTACC
tRNA iMet northern probe	ATCGACCTCTGGGTTATGGGCC
tRNA Tyr 3’ trailer northern probe	CAGACGAAGTCCTTCGAGCC
5.8S rRNA northern probe	GCAAGTGCGTTCGAAGTGTCGATGATCAAT
Readthrough UUA control PCR forward	CGGCAGGAACACAATTACAATTACAGATTGGA
Readthrough UUA control PCR reverse	TCCAATCTGTAATTGTAATTGTGTTCCTGCCG
Readthrough UUG control PCR forward	TCCTTCAACTTCCCTGAGCTCGAAGAC
Readthrough UUG control PCR reverse	TCCAATCTGTAATTGCAATTGTGTTCCTGCCG
