# Supplementary material for: Human DUS1L catalyzes dihydrouridine modification at tRNA positions 16/17, and DUS1L overexpression perturbs translation
Source: Commun Biol. 2024 Oct 2;7:1238. doi: 10.1038/s42003-024-06942-8 (PMC11445529; doi:10.1038/s42003-024-06942-8)
Supplement: Supplementary file 2 — Description of Additional Supplementary Materials [file 42003_2024_6942_MOESM2_ESM.pdf]

Description of Additional Supplementary Files

File name: Dataset 1

Description: Source numerical data for the graphs
